# Outcomes of early relaparotomy in pediatric patients at Tikur Anbessa teaching hospital, Addis Ababa, Ethiopia: a five-year retrospective review

**DOI:** 10.1186/s12893-018-0436-x

**Published:** 2018-11-16

**Authors:** Tihitena Negussie, Abay Gosaye, Belachew Dejene

**Affiliations:** Addis Ababa University, Addis Ababa, Ethiopia

**Keywords:** Pediatrics, Re-laparotomy, Indications, Global surgery, Ethiopia

## Abstract

**Background:**

Early relaparotomy is defined as relaparotomy within the first 30 days following surgery. The aim of this study is to explore the indications, outcomes and factors associated with relaparotomy in our pediatric population.

**Methods:**

We performed a retrospective study of pediatric surgical patients (< 13 yrs.) who underwent relaparotomy at Tikur Anbessa Teaching Hospital between September 1, 2011 and August 31, 2016. All children who had relaparotomy within the first 30 days of the initial surgery were included. We collected patient data including demographics, operative indication, and postoperative outcomes. Data analysis was performed using SPSS Version 23. Chi-square and Fisher’s exact tests were used to report outcomes stratified by patient characteristics. Multivariable logistic regression was used to identify patient variables associated with relaparotomy and other outcomes.

**Results:**

In our patient population, relaparotomy rate was 17.2%. Patient age ranged from 2 days to 12 years with mean age of 37.5 months. Male to female ratio was 1.2:1. Thirty-one (58.5%) relaparotomies were performed between the 5th and 8th postoperative days. The two most common indications for relaparotomy were postoperative intra-abdominal collection and anastomotic leak, accounting for 18 (34.0%) and 17 (32.1%) respectively. Mortality rate following relaparotomy was 26.4%. The most common cause of mortality was sepsis with multi-system organ failure (90.6%). Neonatal age was found to be the independent risk factors for death following relaparotomy, (AOR = 27.59, 95% CI [2.0–379.9]).

**Conclusion:**

Prevalence of relaparotomy in pediatric patients is high (17.2%) in our patient population. Neonatal age was associated with increased mortality following relaparotomy.

## Background

The term relaparotomy is used in multiple contexts in the medical literature to refer to abdominal reoperations [1]. Generally most authors define relaparotomy as operations performed within 60 days after initial surgery [2, 3]. There are several classifications of relaparotomies; early vs. late, planned vs. unplanned, and emergency vs. elective [1]. Early relaparotomy is widely considered to include relaparotomies done within the first 30 days of initial surgery [1].

There is lack of consensus on definition of each type of relaparotomy. Girgoev et al. use the term “urgent relaparotomy” to indicate emergency re-exploration for clinical deterioration, failure to improve, or radiographic evidence of intra-abdominal collection [2]. Oddeke et al. classify relaparotomy as ‘on-demand surgery’ where the patient’s condition necessitates re-exploration and ‘planned’ wherein a relaparotomy is performed at routine intervals for re-exploration, or drainage and peritoneal lavage of the abdominal cavity until findings are negative for ongoing peritonitis [4]. Avedis asserts that outcomes of relaparotomy should be assessed in terms of recovery and of survival, which has been frequently used as an indicator of the quality of medical care [5].

This study is important as it describes the current state of surgical care for pediatric patients in Ethiopia. Number of relaparotomies and outcomes following relaparotomies are known to be an indicator of quality of surgical care. There are very limited literature on this area. Our literature review didn’t reveal any studies that specifically investigate relaparotomy in pediatric populations in African setting. And findings will be useful to inform future research, and in the development of quality improvement initiatives for our pediatric surgical patients. The aim of this study was to explore the indications, outcomes and factors associated with early relaparotomy in our pediatric population.

## Methods

We conducted a retrospective review of all pediatric surgical patients (< 13 yr) who underwent a relaparotomy at Tikur Anbessa Teaching Hospital between September 1, 2011 and August 31, 2016. Tikur Anbessa Teaching Hospital, located in Addis Ababa, Ethiopia, is the primary referral center for all pediatric surgical patients in the country. Annually about 750 emergency and 650 elective general pediatric procedures are performed at Tikur Anbessa.

### Patient selection

All relaparotomy cases during the study period were identified using a combination of operative logbooks, anesthesia and recovery room logbooks. Inclusion criteria included those pediatric surgical patients < 13 years of age who underwent a relaparotomy within the first 30 days of the initial surgery. Patients were excluded if the complete written medical record was unavailable. Data collected included age, sex, address, admission diagnosis, type of surgery, comorbidities, level of the operating surgeon, duration of illness before presentation, presence of peritonitis on presentation, presence of SSI, indications for relaparotomy, number of relaparotomies and outcomes. The primary outcome was postoperative mortality.

Patients were stratified by age groups as follows: neonatal age (< 1 month), 1–12 months, 1–5 years, and > 5 years. Operations performed by a resident without senior surgeon (“consultant surgeon”) involvement were considered as resident cases. All others where senior surgeon was involved were considered consultant cases. Surgical site infection (SSI) was also collected from medical chart review. SSI is diagnosed based on criteria developed by United States Centers for Disease Control and Prevention (CDC). In our study incisional site SSI was analysed alone. So when we say SSI we mean incisional SSI only while organ/ space SSI was included under post-op collection.

### Data analysis

IBM SPSS Statistics for Windows, version 23 (IBM Corp., Armonk N.Y., USA) was used for statistical analysis. Chi-square test was used to report outcomes stratified by patient characteristics. Fisher’s exact test was used when appropriate. Multivariable logistic regression was used to test the influence of variables on the outcome. Variables tested were age, address, type of surgery, admission diagnosis, level of operating surgeon, delayed presentation for more than 48 h, indication for relaparotomy, surgical site infection (SSI) and number of relaparotomy operations. A *p*-value of < .05 was deemed significant.

## Results

### Patient characteristics

A total of 354 laparotomies were performed for pediatric surgical patients between September 1, 2011 and August 31, 2016. There were 61 relaparotomies during the study period. Three cases were excluded due to lack of patient identifiers in the operating room log. Five cases were excluded because charts could not be found for review. Our study population was comprised of 53 relaparotomy cases included in final data analysis. Patient age ranged from 2 days to 12 years with mean age of 37.5 months. Twenty-nine (54.7%) were male while 24 (45.3%) patients were female. With regards to the initial operation, 40 (75.5%) were emergency cases while 13 (24.5%) were elective cases. After the initial laparotomy, patients who required relaparotomy presented with clinical features of peritonitis, intestinal obstruction, complete wound (fascial) dehiscence or stoma-related complications. All relaparotomies in our population were unplanned or emergency operations with indications related to complications from the primary laparotomy. Forty (75.5%) operations occurred during overnight/ weekend duty hours while the remaining 13 (24.5%) operations took place during daytime working hours. Senior residents operated on 43 (81.1%) and the remaining 10 (18.9%) cases had involvement by senior surgeons (consultants) (Table 1). Only 7 (13%) patients presented to the hospital within 48 h of symptom onset during the initial laparotomy. Six of these patients were patients operated on emergency basis.

**Table 1:** Showing socio-demographic characteristics of the study participants and their presenting surgical conditions

Characteristics	Number (%)
Sex	
Male	29 (54.7%)
Female	24 (45.3%)
Address	
Addis Ababa	22 (41.5%)
Out of Addis Ababa	31 (58.5%)
Surgery Classification	
Emergency	40 (75.5%)
Elective	13 (24.5%)
Peritonitis (on presentation)	
Yes	11 (20.8%)
No	42 (79.2%)
Trauma	
Yes	4 (7.5%)
No	49 (92.5%)
Age	
< 1 month	10 (18.9%)
1–12 months	16 (30.2%)
1 yr.- 5 yrs.	12 (22.6%)
> 5 yrs.	15 (28.3%)
Operating surgeons	
Senior Resident	43 (81.3%)
Consultant surgeon	10 (18.7%)

#### Indications

Timing of relaparotomy ranged from the second postoperative day to the 27th postoperative day. Thirty-one (58.5%) cases of relaparotomy were performed between the 5th and 8th postoperative days. The two most common indications for relaparotomy were postoperative intra-abdominal collection and anastomotic leak, accounting for 18 (34%) and 17 (32.1%), respectively.

Other indications included complete wound dehiscence (7, 13.2%), stoma revision (5, 9.4%), postoperative adhesions (5, 9.4%) and anastomotic stricture (1, 1.9%). Twelve (22.6%) of the patients had associated surgical site infection (Table 2).

**Table 2:** Comparing indications for relaparotomy and other postoperative complications with mortality rate

Post-operative complications	Frequency (%)	Mortality rate (%)
Indications for relaparotomy		
Anastomotic leak	17(32.0)	6(35.3)
Post-op collection	18(33.9)	3(16.6)
Complete wound dehiscence	7(13.2)	3(42.8)
Stoma revision	4(7.5)	2(50)
Post-op adhesion	5(9.4)	0(0)
Parastomal hernia	1(1.9)	0(0)
others	1(1.9)	0(0)
Other associated complications		
SSI	12(22.6)	2(16.6)

Intussusception and appendicitis were the two major indications for initial laparotomy. They account for 15 (28.3%) and 11 (20.8%), respectively. Other major admission diagnoses included trauma, intestinal atresia, colostomy for Hirshsprung disease (HSD) and ano-rectal malformation (ARM), and pull-through procedures for HSD.

#### Outcomes

Overall mortality following relaparotomy was 26.4%. Fourteen patients died postoperative while thirty-nine (73.6%) survived to discharge. Eleven (78.6%) of the deaths occurred during the first 48 h following the relaparotomy. The most common cause of death was sepsis with multi-organ dysfunction (MOD). Death from MOD occurred in 9 patients (90.6%), fluid and electrolyte disturbance in one patient (1.9%) and was unknown in four (7.5%) of the cases.

Postoperative mortality after relaparotomy for intussusception, was 33.3% (Five out of fifteen cases). All operations for intussusception requiring relaparotomy were initially performed by senior residents during duty hours. Similarly, all laparotomies for appendicitis were performed by residents, but mortality for these patients was 0%.

#### Factors associated with mortality following relaparotomy

Univariate logistic regression analyses were performed for all variables as potential predictors of death following relaparotomy. All variables selected as potential predictors of death after relaparotomy were included in multivariate analysis. These included type of operation (emergency vs. elective), delayed presentation more than 48 h, level of operating surgeon, number of relaparotomy operations, surgical site infection and age category. The significant result is bolded. (Table 3). In multivariate analysis, neonatal age (< 1 month) was found to be an independent risk factor for death following relaparotomy, (AOR = 27.59, 95% CI [2.0–379.9]).

**Table 3:** Results of univariate and multivariate analysis

Variables	Univariate *(*P*-value)	Multivariate (AOR)	95% CI
Types of operation (Emergency vs. Elective)	.754	0.75	[.03–17.54]
Level of operating surgeon	.611	4.63	[.11–189.34]
Number of relaparotomies	.285	0.20	[.02–2.17]
SSI	.384	0.85	[.12–6.21]
Delayed presentation more than 48 h	.300	3.35	[.44–25.34]
Age category *	**.013**	**27.59**	**[2.00–379.91]**

*Bolded is the significant result

## Discussion

To our knowledge, this is the first study describing the prevalence of relaparotomy and subsequent outcomes in a pediatric surgical patient population in Africa. Our relaparotomy rate of 17.2% is similar to few studies done on outcome of neonatal surgery mainly for patients with intestinal atresia in developed country, which reported relaparotomy rate ranging from 14.5 to 17% [6, 7]. Kumaran et al. reported overall relaparotomy rate of 14.5% [6] done only for the indications of adhesive obstructions while Piper et al. reported 17% of relaparotomy rate [7].

Only 7 (13%) patients presented to the hospital within 48 h of symptom onset. We found no statistically significant association between delayed presentation more than 48 h after onset of illness and outcome after relaparotomy. Although delayed presentation might affect the outcome of initial laparotomy, we found no association with outcome of relaparotomy.

In our study, the two major indications for relaparotomy were postoperative intra-abdominal fluid collection and anastomotic leak. In our clinical setting without readily available radiographic imaging capabilities, these diagnoses are generally made intra-operatively with varied preoperative manifestations of peritonitis. Our results are similar to previous studies on indications for relaparotomy in the adult patient population. In Russia, the most common indications for relaparotomy included peritonitis (51.3%), ileus (30.2%), eventration (7.2%), hemorrhage (7.9%) and other causes (3.3%) [8]. In a series of 121 relaparotomies in 16,719 cases of laparotomy in Sweden, indications for relaparotomy have included peritonitis (32%), ileus (25%), wound rupture (22%), hemorrhage (19%), and other causes (2%) [9]. In studies done on pediatrics shows postoperative abdominal evisceration in children accounts for 0.2–1.2% and is one of the indication for relaparatomy [10, 11].

Postoperative fluid collections and anastomotic leak comprised approximately 66.1% of indications for relaparotomy. The study done by Kumaran et al. reported also other complications like anastomotic leak and stenosis and stoma complications that potentially requires reoperation in addition to adhesive obstruction although it clearly didn’t mention whether reoperation was done or not [7]. In higher resource settings the typical management algorithm for intra-abdominal fluid collection, abscess, or contained anastomotic leak includes image-guided percutaneous drainage by Interventional Radiology. This is not currently available in our routine practice, however improved diagnostic and procedural capabilities of Interventional Radiology may significantly reduce need for reoperation and possibly improve patient outcomes [12, 13].

In our study the postoperative mortality rate was 26.4%. This figure is very similar to studies conducted in Sweden and Russia, which found mortality rate of 28.1 and 26%, respectively [8, 9]. And those patients who were managed for wound dehiscence in the above literatures has mortality rate of 11 and 12.5% [10, 11]. The majority (90.6%) of deaths following relaparotomy were attributed to multisystem organ dysfunction due to sepsis. These findings are also similar to one adult study in India that found mortality rate after relaparotomy to be 33.2% with sepsis and multi-organ failure being the commonest cause of death [14].

We found significant association between neonatal age and mortality after relaparotomy (*p* = 0.013). The seven neonates with an admitting diagnosis of intestinal atresia had the highest mortality rate at 71.4%, though intestinal atresia was not an independent predictor of mortality (*p* = 0.999).in our multivariate analysis. We hypothesize that the neonatal physiology itself is a risk factor as there were no concomitant diseases identified in any of this patient subset. Gestational age was not known from the patient record, therefore we were unable to determine if diseases of prematurity may have contributed to mortality. In a previous study of neonatal surgical admission outcomes at our hospital, there was an overall mortality of 24.4% [15]. In a systematic review of outcomes following neonatal surgery in Africa, showed that neonatal mortality rate was 36·9% before 2005 and *29*·1% between 2005 and 2014. *p* < 0·001), but mortality did not vary between the groups for similar neonatal conditions. The major documented challenges were delayed presentation and inadequate facilities for perioperative care in 39 (76·5%) studies, dearth of trained support personnel in 32 (62·7%), and absence of neonatal intensive care in 29 (56·9%) [16]. The challenges mentioned applies in a set-up like ours.

Mortality was also high (33.3%) for patients who required reoperation for intussusception. This result is consistent with many reports from Africa. Intussusception is a leading cause of intestinal obstruction in children. One study from Nigeria reported 29% of intestinal obstruction from intussusception [17]. Mortality rates of intussusception in sub-Saharan Africa vary widely and have been reported at 0–55% [18]. The commonest cause of intestinal obstruction in a study in India was also intussusception with 34% of obstruction related to intussusception [19].

Appendicitis was the second most common admission diagnosis requiring relaparotomy with 11 (20.8%) patients. Acute appendicitis is a common cause of acute abdomen in children worldwide. In Ethiopia, appendicitis accounts for 12% of emergency pediatric surgery and in the Congo, it constitutes 30% of pediatrics gastrointestinal surgery [20, 21]. Mortality from appendicitis can be up to 4% in some sub-Saharan Africa settings [22]. Fortunately, in our study, no patients died following relaparotomy in those with an admitting diagnosis of appendicitis.

Furthermore, patients who had anastomotic leak as an indication for the relaparotomy had a higher mortality rate (35.3%). This result is also similar with other literature [23, 24]. Although surgical site infection rate was high, it was not significantly associated with increased mortality. The same is true in other studies as well. Surgical site infections have been found to increase morbidity and hospital length of stay, however according to Joseph et al., there is no associated increase in mortality [25].

As Tikur Anbessa is an academic center, with all Ethiopian surgical residents rotating through this pediatric surgical unit, there is much to be learned and implemented from these results. Although the association of the operating surgeon with the outcome of the relaparotomy was not statistically significantly, likely due to small sample size, we found that the majority of patients requiring relaparotomy had their initial operation performed by senior residents (75.5%), and during duty hours (75.5%). It is difficult to assess the specific differences in operations performed by senior consultants and residents because typically types of cases performed by consultants and residents differ. The high rate of relaparotomy is likely multifactorial including technical failures or patient disease severity. In this residency-training program, residents treat most emergency conditions including intussusception and appendicitis alone. While resident autonomy is an important part of training, our findings highlight that perhaps increased supervision for emergency operations performed during duty hours would result in fewer reoperations. Prospective studies should be considered to assess the effect of resident supervision on patient outcomes in this setting. In light of these results the trend is changing now.

Our study showed higher mortality rate (35.3%) in those patients who had anastomotic leak as an indication for the relaparotomy. This result is also similar with other literatures [23, 24]. Among the 12 patients who had SSI only 2 of them died post relaparotomy. The association of SSI and relaparotomy outcome is not statically significant in our study. The same is true in other studies even if the morbidity and hospital stay of patients with surgical site infection increases; there is no associated mortality with it [25].

**Fig. 1:**
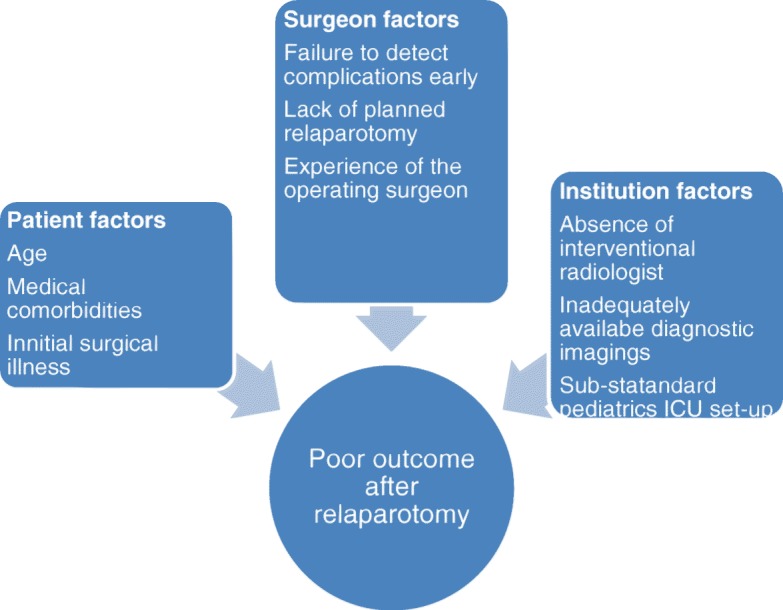
Flow chart: Showing factors contributing to poor outcomes following relaparotomy

To bring improvement in quality of care one should work on addressing patient factors, surgeons’ factors and institution factors listed in the flow chart above (Fig. 1).

## Limitations

- Single center study with small number of patients making our findings difficult to generalize to other centers.
  - ○ Though provides an important baseline for future work within resource-constrained settings like that of sub-Saharan Africa such as ours.
- Retrospectively reviewed
  - ○ Helps provide foundation for a future prospective study to identify modifiable risk factors, which can be modified to improve surgical quality in our setting.
- Mortality rates for intussusception unknown as only studied those requiring relaparotomy.

## Conclusion

In our pediatric surgical population, relaparotomy rate and associated mortality is high, particularly in neonates. These findings provide a baseline for a future prospective study to determine other important modifiable risk factors for poor patient outcomes in resource-constrained settings.

### Recommendation

Additionally, given the complex pediatric physiology, we recommend that pediatrics surgical cases should be supervised by experienced surgeon like senior consultant or pediatric surgery fellow.

## Abbreviations

ARM: Ano-rectal malformation
HSD: Hirshsprung disease
MOD: Multi organ dysfunction
SSI: Surgical site infection
